# Vancomycin Flight Simulator: A Team-Based Learning Exercise

**DOI:** 10.3390/pharmacy11010013

**Published:** 2023-01-11

**Authors:** Nicholas W. Carris, Jaclyn D. Cole, Ann Snyder Franklin, Katlynd M. Sunjic

**Affiliations:** 1 Taneja College of Pharmacy, University of South Florida, 12901 Bruce B. Downs Blvd MDC 30, Tampa, FL 33612, USA; 2 Southwestern Vermont Medical Center, 100 Hospital Drive, Bennington, VT 05201, USA

**Keywords:** education, team-based learning, pharmacokinetics, vancomycin, patient monitoring, therapeutic drug monitoring

## Abstract

BACKGROUND: Team-based learning (TBL) encourages learners to think critically to solve problems they will face in practice. Pharmacokinetic dosing and monitoring are complex skills requiring the application of learned knowledge. The study sought to assess the impact of a TBL, vancomycin dosing activity in a Pharmaceutical Skills IV course measured with exam question performance during the second professional year. METHODS: This retrospective, descriptive study relates a TBL activity, assigned to 85 students, which included an individual student pre-preparation quiz, assigned readings, in-class individual and team-based readiness assessments, small group application of a vancomycin patient case, and group discussion/feedback on clinical decisions with supportive reasoning. The class year before and class year of the TBL implementation were compared using the total percentage of points possible earned by the class years, by topic. To minimize potential confounding, the primary outcome was the change in topic performance by the rank difficulty (e.g., the largest possible benefit being the hardest topic becoming the easiest with no other variation in topic rank difficulty). RESULTS: In the year of implementation, the mean individual readiness assurance test (IRAT) performance was 5.5 ± 1.88 (10 points possible, 55%). The mean team readiness assurance test (TRAT) performance was 10 of 10 points possible (100%). The class exam item performance in the year before (*n* = 101) and year of (*n* = 84) TBL implementation showed a general decline in exam scores. However, the vancomycin topic difficultly went from fifth easiest, to second easiest, with less than 1% change in raw score. CONCLUSIONS: Implementation of a pharmacokinetic TBL activity appeared to moderately support the students’ vancomycin learning. Additional studies are warranted on APPE readiness and performance.

## 1. Introduction

Pharmacokinetics encompasses multiple complex concepts and skills taught in the Doctor of Pharmacy (Pharm.D.) curricula. The Accreditation Council for Pharmacy Education’s 2016 standards require education on clinical pharmacokinetics, noting learners’ need to calculate appropriate doses and adjust therapy for safety and outcomes [1]. Variation exists in how clinical pharmacokinetics is taught across colleges of pharmacy, suggesting that process improvements are possible [2]. Additionally, there can be multiple reasonable therapeutic options, yet course work involving a mathematical calculation often directs students to a single correct answer. Team-based learning (TBL) is one educational method which can promote discussion, critical thinking, and timely feedback to engage learners in complex decision-making skills.

The main goal of TBL is to scaffold student learning in a manner requiring the application of taught material to address real-world issues through critical thinking [3]. TBL may improve transferable skills through peer learning and academic performance [4,5,6,7]. TBL includes four core components: assignment design, group assignment, accountability, and feedback [8]. TBL assignments require the same problem to be provided to all groups. Learner accountability is often tracked through an Individual Readiness Assurance Test (IRAT) followed by a Team Readiness Assurance Test (TRAT). Feedback for testing and TBL performance is provided in real time. High-quality TBL activities in pharmacy education should use authentic pharmacy challenges/situations regarding relevant pharmacy tasks, which encourage rich discussion and provide effective feedback to the groups [9].

Pharmaceutical Skills courses provide students the opportunity to apply materials learned in other courses. In an effort to enhance pharmacokinetics learning, a vancomycin dosing TBL case series was added to a second-year Pharmaceutical Skills IV course (“Skills”), aligned with the simultaneously offered Clinical Pharmacokinetics/Pharmacodynamics II (“cPKPD”) course. Vancomycin dosing is a common clinical pharmacokinetics consultation. The primary course content for vancomycin dosing was provided in the cPKPD course in the Spring of the second year of the Pharm.D. curriculum at the time this learning activity was implemented. The vancomycin dosing content consisted of estimating renal function, estimating volume of distribution, estimating vancomycin elimination rate, determining initial vancomycin doses and regimens, monitoring the appropriate therapeutic drug, adjusting doses, and pulse dosing. In the cPKPD class, students received practice problems and clinical vignettes (more lengthy, multi-step clinical scenarios) during class (multiple per lecture hour), followed by a homework problem set for students to solve that was concurrent with the module content (provided at the start and due just prior to the exam day). Given this structure, the assigned homework was not graded prior to the exams. However, students were familiar with case-based questions before the Skills TBL activity. The report herein describes the TBL activity in Skills and the learning outcomes assessed in the cPKPD course.

## 2. Materials and Methods

The study objective was to assess the impact of a newly implemented TBL activity within the Skills course on student performance, between two class years, on related material on cPKPD exams. The University of South Florida Institutional Review Board waived review.

**Figure 1:**
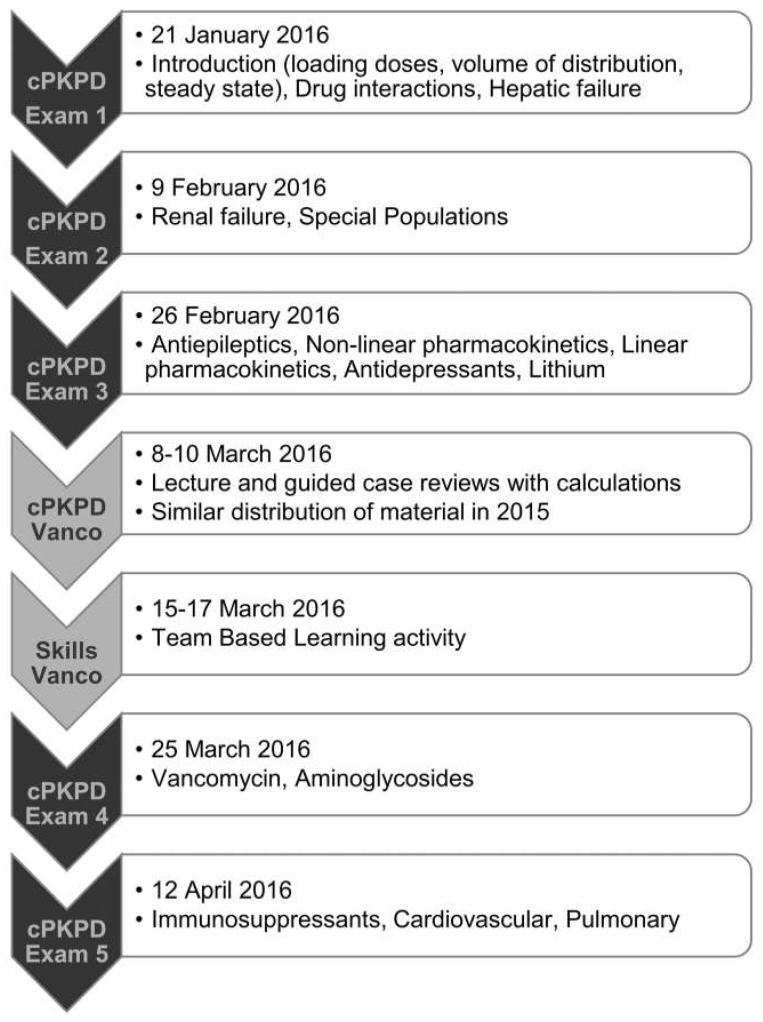
Time Sequence of Vancomycin Activity with Clinical Pharmacokinetics and Pharmacodynamics (cPKPD) Course †. † course topics were the same between years.

The TBL activity was developed collaboratively by the Skills and cPKPD course coordinators with feedback from a TBL expert [10]. The cPKPD course was a 3-credit hour course, with up to 6 h dedicated to teaching vancomycin content—at least 3 h of lecture, with up to 3 h of working problem sets with vancomycin dosing calculations and related clinical scenarios. The course activity sequence is shown in Figure 1 with the cPKPD exam covering vancomycin occurring 8 or 10 days after the vancomycin TBL activity depending on which class section the students attended. It was the students’ first exposure to TBL as part of the standard Pharm.D. curriculum. The activity materials (Appendix A, Appendix B, Appendix C, Appendix D, Appendix E and Appendix F) are from 2020, minimally modified from the 2016 materials. Changes address typographical errors, clarity in language, and removal of discussion regarding D-test and interpreting culture data. The TBL activity was designed to communicate the clinical relevance and importance of safe and effective vancomycin dosing and required learner accountability at each step (i.e., IRAT, TRAT, verbal defense).

For in-class, small-group work, 85 students worked with their assigned “class groups” which met Tuesday or Thursday. Groups included six to eight students. Two students were absent for the activity. Two students typically scheduled for Tuesday were granted permission to attend the Thursday session and were integrated into an existing group. The in-class TBL activity was led by the skills coordinator during a 2-h and 50-min class period in a large-group learning room. The cPKPD course coordinator attended class sessions to answer questions pertaining to alignment of course material and to relate it to the upcoming cPKPD exam.

The focus of the TBL activity was application of previously learned materials for patient assessment, critical thinking, and clinical decision-making. Calculations were required to complete the activities; however, the primary focus remained on interpretation of the calculated values (Appendix D). A vancomycin dosing protocol was developed and provided to ensure groups used identical methods of dosing to create consistency in calculated values (e.g., all vancomycin doses ≤ 2 g administered over 2 h) to ensure the discussion was streamlined toward patient assessment and clinical decisions rather than calculations (Appendix F).

The learning objectives of the TBL activity were for students to be able to 1) assess a patient case to determine the best application of estimated renal function, 2) select an appropriate initial vancomycin dose (regimen or one-time dose), and 3) establish a monitoring plan based on changing renal function to determine future dose. Prior to the in-class TBL activity, students completed a “pre-preparation quiz” (Appendix A) to assess learned material from cPKPD. Students were assigned two readings to facilitate their preparation and a list of values to be able to calculate (Appendix B).

At the beginning of class, students individually completed an IRAT (~10 min) and then completed the same assessment as a group, TRAT (~5 min) (Appendix C). The skills coordinator led a quiz debrief and emphasized the purpose and organization of the TBL activity (~10 min). Next, the TBL activity moved into group participation on the case series (~2-h and 25-min) (Appendix D). The TBL activity followed a pharmacokinetic consult for vancomycin in a single patient during an inpatient stay over five “patient case-days”. Groups reviewed the patient’s presentation, made assessments, and chose clinical action. For each patient case-day, groups had ~15–20 min to work up the case. During this time, groups decided on their preferred answer to each question. Groups were held accountable to their preferred answer by holding up a response card for the discussion question being addressed [11]. When called on, groups verbally defended the reasoning for their answer. Following discussion, the instructor clarified, confirmed, and summarized the salient points relating back to the activity learning objectives. This process repeated for all questions for each case-day.

Following the activity, an anonymous survey was distributed (Appendix E) assessing general opinions about the activity, not related to a specific learning outcome, and, therefore, it was not formally validated. The components of the TBL activity were graded for participation: pre-preparation quiz (20% of activity grade), IRAT (10% of activity grade), TRAT (20% of activity grade), TBL patient case activity (30% of activity grade), and end of activity survey (20% of activity grade).

Vancomycin dosing summative assessments occurred in cPKPD course exams. To avoid bias from retrospectively comparing raw exam scores on non-identical exams (2015 versus 2016), we compared percent of points possible earned by the class by topic between years. To complete this comparison between class years, the question topic and question type of every exam question on all cPKPD course exams for each course year were assessed. In both years, exams were composed of the same question types: multiple choice, true–false, fill in the blank, and essay. Then the percents of points possible earned by the class years were compared between years, by question topic. As such, the topic with the highest percent of points possible earned by an entire class year, on all associated questions, would be considered the easiest topic. Whereas the topic with the lowest percent of points possible earned by the entire class year would be considered the hardest topic. The primary outcome was change in topic performance by rank difficulty, to minimize potential confounding. Assessing student learning by question topic area assumes that, in general, harder topics remain harder and easier topics remain easier. This approach is less prone to bias compared to the assumptions required for comparing raw exam scores (i.e., that student ability and exam difficulty are the same year over year).

Students without exam data for all exams assessed during the study period were removed from the data set, as were questions on cPKPD exams with missing data or which were dropped from the exam score or had all responses accepted as correct. As a retrospective study, determination to exclude questions in the final grade (e.g., dropped questions) were not performed in a protocolized fashion, though course coordinators evaluated question quality and item performance. As such, all questions meeting the criteria above were included in the present analysis. Prior to the study results being reviewed by the cPKPD course coordinator, that course coordinator assessed the cPKPD course for changes in topic instructors, instruction methods, and content hours. Survey data were manually entered into REDCap electronic data capture tools hosted at the University of South Florida [12,13].

## 3. Results

The final data set for analysis included 101 students from year 1, with 4 excluded for missing exam data, and 84 students from year 2, with 1 excluded for missing exam data. Four of the five Pharmacokinetics exams were assessed. Exam 1 was excluded from both years due to missing data. Over four exams in year 1, 149 questions were assessed. Eighteen questions were excluded for not being scored. Across all topics in year 1, a total of 291.8 points were possible on the assessed questions. Over four exams in year 2, 130 questions were assessed. One question was excluded for not being scored. Across all topics in year 2, a total of 332.18 points were possible on the assessed questions. Two students in year 2 did not participate in the TBL activity. One student’s exams scores were included in the analysis. One student was excluded for incomplete exam data. The only major change identified in the cPKPD course was a change in the instructor teaching theophylline and digoxin content (Table 1).

The mean ± standard deviation score on the pre-preparation quiz before the TBL activity was 3.9 ± 1.06 (5 possible points, 78%). The mean IRAT performance was 5.5 ± 1.88 (10 points possible, 55%). Questions with relatively lower performance (<50% correct) on IRAT were calculation-based (questions 2, 6, 7, 8) rather than conceptual knowledge-based. One exception was that 37% of students answered question 10 correctly, which regarded a pre-reading topic addressing when to proactively change a vancomycin regimen before obtaining a first trough. The IRAT score did not differ between class days (mean ± standard deviation; day-one 5.45 ± 1.92, day-two 5.56 ± 1.90; *p* = 0.80). The mean TRAT score on the only attempt was 10 out of 10 points.

Overall, the data demonstrate a year-over-year decrease in raw exam scores. By topic, the largest single improvement in rank topic performance was warfarin (Table 2). However, this was matched with a decline in warfarin interaction performance. A large improvement in the rank topic performance was observed in heparin, followed by vancomycin. Neither heparin nor vancomycin showed a meaningful absolute increase in the percent of points possible earned on the raw exam scores (both <1% absolute increase). Therefore, their change in rank is related to the maintenance of the percent of points possible earned, and their relative rank improvement related to the number of topics with a year-over-year decrease in percent of points possible earned. When restricting the analysis to topics with at least eight questions in each year (Table 3), the trend of decreased raw exam scores remained. Similarly, heparin and vancomycin remained the topics with the largest rank improvement. Notably, while vancomycin demonstrated a maintenance in the percent of points possible earned year over year, there was a large decrease in aminoglycoside’s percent of points possible earned year over year. No discernable trend was identified by Bloom’s taxonomy or question type.

Both increases and decreases in the proportion of recall-based questions co-occurred with increases and decreases in topic performance. Similarly, both increase and decreases in the proportion of multiple-choice questions co-occurred with increases and decreases in topic performance (Data not shown). Survey data demonstrated a positive view on the group dynamics and interdependence (Table 4). The majority of students either strongly agreed or agreed that their abilities improved regarding applying pharmacokinetic concepts, understanding of renally cleared medications, how renal function and volume of distribution affects vancomycin dosing, and linear pharmacokinetics.

## 4. Discussion

Overall, this study suggests moderately improved learning with an aligned TBL activity between the Skills course and the cPKPD course. While raw scores did not improve, the a priori analysis method accounted for this by comparing topic performance between years. In support of our conclusion of improved vancomycin learning with the vancomycin dosing activity, there was a notable decline in aminoglycoside performance, while vancomycin performance was maintained. The decrease in aminoglycoside performance is in line with the general decline in raw exam scores year over year. Potential reasons aminoglycosides did not see a corollary benefit related to the enhanced education on vancomycin may be due to 1) no additional aminoglycoside practice problems, 2) the multiple different dosing strategies with aminoglycosides (e.g., Hartford nomogram), and 3) the dose adjustments with aminoglycosides versus vancomycin in the presence of renal dysfunction being dissimilar.

Overall, the activity reinforced the pharmacokinetic skills taught in cPKPD. The alignment between courses and use of TBL seemed to support student learning and abilities in a complex, variable topic. This is an important application opportunity, as safe and effective pharmacokinetic dosing cannot rely on rote memorization and requires experience and application to support learners’ abilities, skills, and confidence. Importantly, the results of the present study are in line with prior assessments of including TBL in pharmacokinetic course work [14,15]. In addition to case-based questions, immediate feedback given after the TRAT and case discussions assesses students’ mastery of course outcomes, encourages deep learning and critical thinking skills, and is preferred by students [15]. One eight-year retrospective review identified that increasing amounts of active learning increased student performance, despite the potential for decreased student evaluations [14]. Similarly, another study identified that multiple strategies for including case-based learning resulted in improved exam scores compared to more traditional teaching methods [15]. Additionally, the results being in line with prior research on active learning points to a strength of our study design—that being, that rank difficultly as an assessment of performance following a targeted intervention may be more able to identify differences (compared to raw exam score comparisons) in a pre–post retrospective study.

There are some limitations to the study and the interpretation of the results. While the score on the pre-preparation quiz (78%) suggests reasonable baseline knowledge following the cPKPD course work, the mean IRAT performance (55%) suggests a low level of preparation specific to the TBL activity, potentially relating to how points were earned (completion). However, the high performance on TRAT suggests the opportunity for peer teaching and activity readiness. Moreover, the enhanced learning observed regarding vancomycin is consistent with the study’s survey results and prior reports of enhanced student performance [6,7]. Additionally, the cohorts were not matched, and there was variation in the exam performance between class years. The data analysis strategy accounted for varying ability between cohorts, somewhat, and the differing ability between class years is not generally unexpected and, further, was in line with class metrics [16]. The analysis strategy assumes that topic difficulty remains similar year over year. In support of this assumption, 50% of topics had a change in rank ≤ 2. Additionally, the analysis only included two class years, and, as such, inferential statistics were not possible. There were no controls over teaching methods between years, though content changes were minimal. Additionally, there were no controls on student workload, effort on other courses, or effort on outside activities. It is possible that the ratio of students in inpatient versus outpatient internship experiences changed year over year. However, we would not expect this to impact results. We do not anticipate interns in the second professional year at our local institutions to be involved in vancomycin dosing and monitoring. While the results do not form robust support for this TBL activity, the results could have been impacted by differences in the exams given between years. While the cPKPD exams were not identical between years, the formats were consistent, though variation in difficultly could have existed. There was a decrease in the number of exam questions covering vancomycin which could have impacted results by offering students differing chances to answer correctly. However, identical exams would not have eliminated the potential for bias as identical exams would be subject to the potential for students to communicate exam content between class years. One of the five exams was not analyzed. However, the excluded exam (exam 1) consisted primarily of general pharmacokinetic and pharmacodynamic topics and the associated introductory clinical considerations. Therefore, we expect its exclusion is less likely to affect the overall results than exclusion of a different exam would. Additionally, the end of unit exam performance is also an indirect measure of the value of a given activity. The students appeared to value the activity (Table 4) which provides information on student perceptions of learning/engagement which are important for student processing of new information. However, Likert scales are highly subjective, and these results are secondary to the changes in test scores. Finally, the data of the present study pre-date the COVID-19 pandemic, and changing teaching methods and student populations may diminish replicability.

## 5. Conclusions

Overall, implementation of a pharmacokinetic TBL activity within the skills course appeared to moderately support the understanding of vancomycin assessment and dosing introduced in the cPKPD course during the second year of a four-year Doctor of Pharmacy program. In subsequent years, the activity has been refined, and the approach to this activity was expanded to outpatient warfarin consults with ease, given the adaptable format of the TBL activity (Appendix A, Appendix B, Appendix C, Appendix D, Appendix E and Appendix F). Additional study is warranted on how the TBL activity relates to APPE readiness and the potential to host the activity again later in the semester or in the following academic year to further promote retention and APPE readiness.

## Figures and Tables

**Table 1:** Identified Difference † is Content Delivery Between years (Exams 2–5) in Clinical Pharmacokinetics/Pharmacodynamics II Course.

Topic	Change in Primary Topic Teacher	Year 1 Teacher Was Trainee	Year 2 Teacher Was Trainee	Major Change in Methods	Change in Classroom Content Hours
Warfarin	No	No	No	No	No
Theophylline	Yes	No	No	No	No
Heparins/LMWH	No	No	No	No	No
Vancomycin	No	No	No	No	No
Special Populations (e.g., age, organ related)	Yes	No	Yes ‡	No	No
Antidepressants	No	No	No	No	No
Antidepressant Interactions	No	No	No	No	No
Digoxin	Yes	No	No	No	No
Other Pharmacokinetics Pharmacodynamics	No	No	No	No	No
Immunosuppressants	No	No	No	No	No
Warfarin Interactions	No	No	No	No	No
Aminoglycosides	No	No	No	No	No
Antiepileptic Drugs	No	No	No	No	No
Drug Interactions (general)	No	No	No	No	No

† Course schedules and syllabi were reviewed by the Clinical Pharmacokinetics/Pharmacodynamics II course coordinator prior to knowledge of the study’s results. ‡ Fourth-year pharmacy student under the supervision of faculty.

**Table 2:** Comparison of Topics Between Years.

Topic	Number of Questions (Year 1)	Percent Points Earned (Year 1)	Topic Rank (Year 1)	Number of Questions (Year 2)	Percent Points Earned (Year 2)	Topic Rank (Year 2)	Change in Rank	Change in Percent of Points Earned
Theophylline	1	83.33	3	1	88.10	1	2	4.76
Vancomycin	18	81.93	5	10	82.26	2	3	0.33
Warfarin	3	73.86	10	2	81.75	3	7	7.89
Special Populations (e.g., age, organ related)	26	79.71	6	37	79.00	4	2	−0.71
Antidepressants	4	79.41	7	9	72.55	5	2	−6.86
Heparins/LMWH	14	69.92	13	17	70.88	6	7	0.96
Aminoglycosides	27	91.10	1	14	70.25	7	−6	−20.85
Antiepileptic Drugs	8	89.75	2	12	68.45	8	−6	−21.30
Antidepressant Interactions	3	74.18	9	3	67.06	9	0	−7.12
Digoxin	3	71.57	11	2	64.29	10	1	−7.28
Warfarin Interactions	3	82.03	4	1	63.10	11	−7	−18.93
Immunosuppressants	9	70.83	12	11	58.96	12	0	−11.88
Other Pharmacokinetics Pharmacodynamics	15	61.03	14	9	53.52	13	1	−7.51
Drug Interactions (general)	15	77.97	8	2	45.94	14	−6	−32.04

**Table 3:** Comparison Between Years of Topics with at Least Eight Questions in Each Year.

Topic	Number of Questions (Year 1)	Percent Points Earned (Year 1)	Topic Rank (Year 1)	Number of Questions (Year 2)	Percent Points Earned (Year 2)	Topic Rank (Year 2)	Change in Rank	Change in Percent of Points Earned
Heparins/LMWH	14	69.92	6	17	70.88	3	3	0.96
Vancomycin	18	81.93	3	10	82.26	1	2	0.33
Special Populations (e.g., age, organ related)	26	79.71	4	37	79.00	2	2	−0.71
Other Pharmacokinetics Pharmacodynamics	15	61.03	7	9	53.52	7	0	−7.51
Immunosuppressants	9	70.83	5	11	58.96	6	−1	−11.88
Aminoglycosides	27	91.10	1	14	70.25	4	−3	−20.85
Antiepileptic Drugs	8	89.75	2	12	68.45	5	−3	−21.30

**Table 4:** Survey Results (*n* = 81), data reported as *n* (%).

Rating	Consistently	Regularly	Occasionally	Rarely	Never	Unanswered
My team contributes to team meetings to achieve group tasks	64 (79)	15 (19)	1 (1)	0 (0)	0 (0)	1 (1)
My team maintains positive group communication	62 (77)	17 (21)	1 (1)	0 (0)	0 (0)	1 (1)
My team displays a positive attitude	60 (74)	18 (22)	1 (1)	0 (0)	0 (0)	2 (2)
Rating	Strongly agree	Agree	Neutral	Disagree	Strongly Disagree	Unanswered
The team worked best when we coordinated our work closely	57 (70)	21 (26)	0 (0)	1 (1)	1 (1)	1 (1)
Team members had to work together to complete group tasks	52 (64)	23 (28)	5 (6)	0 (0)	1 (1)	0 (0)
The way individual members performed their jobs had a significant impact on others in the team	55 (68)	22 (27)	3 (4)	0 (0)	1 (1)	0 (0)
My ability to apply pharmacokinetic concepts in establishing a therapeutic regimen for vancomycin has improved	34 (42)	36 (44)	4 (5)	5 (6)	2 (2)	0 (0)
My understanding of medications that are renally cleared has improved	34 (42)	33 (41)	8 (10)	6 (7)	0 (0)	0 (0)
My understanding of how renal function and volume of distribution affects vancomycin dose has improved	38 (47)	30 (37)	7 (9)	6 (7)	0 (0)	0 (0)
My understanding of linear pharmacokinetics has improved	39 (48)	28 (35)	7 (9)	7 (9)	0 (0)	0 (0)

## Data Availability

No data are available from this study.

## Appendix A. Pre-Preparation Quiz

Pre-preparation and Pre-class quiz

Take this quiz prior to preparing for this week’s activities. This quiz is graded for completion.

1. In a patient that is male, 85 kg, 5′10” tall, you should use which weight to dose vancomycin?
  - Ideal body weight
  - Adjusted body weight
  - Total body weight
2. In a patient that is male, 85 kg, 5′10” tall, you should use which weight to calculate creatinine clearance?
  - Ideal body weight
  - Adjusted body weight
  - Total body weight
3. If Ke = 0.099, what is the estimated half-life?
  - 6 h
  - 7 h
  - 8 h
4. If 1000 mg of a drug was given I.V. push in a patient that is 100 kg, assuming a volume of distribution of 0.7 L/Kg, what would be the expected Cmax?
  - 12.29 mcg/mL
  - 13.29 mcg/mL
  - 14.29 mcg/mL
5. For a fixed dose of a drug, if a patient’s volume of distribution increases, the concentration of the drug achieved in the body…
  - Increases
  - Decreases
  - Remains the same

## Appendix B. Required Preparation

**Objectives**

At the end of this exercise, if given a case of moderate complexity or less, students should be able to:

1. Assess a patient case to determine if renal function estimates are reliable
2. Select an initial vancomycin dose (regimen or one time dose)
3. Establish a monitoring plan (including timing) regarding renal function, vancomycin concentration, and vancomycin dose.

**Pre-class instructions**

1. Take the pre-quiz assigned in CANVAS individually. You will have one attempt. Your performance on the quiz will not count toward your grade. However, completion of the quiz will count toward your “participation grade” for the week’s activities. This quiz is required to be completed prior to class and should be complete prior to engaging in the preparation material/readings below.
2. Read and understand the following articles:
  - Rybak M, Lomaestro B, Rotschafer JC, Moellering R Jr, Craig W, Billeter M, Dalovisio JR, Levine DP. Therapeutic monitoring of vancomycin in adult patients: a consensus review of the American Society of Health-System Pharmacists, the Infectious Diseases Society of America, and the Society of Infectious Diseases Pharmacists. Am J Health Syst Pharm. 2009 Jan 1;66(1):82-98. doi: 10.2146/ajhp080434.
  - Jung Y, Song KH, Cho Je, Kim HS, Kim NH, Kim TS, Choe PG, Chung JY, Park WB, Bang JH, Kim ES, Park KU, Park SW, Kim HB, Kim NJ, Oh MD. Area under the concentration-time curve to minimum inhibitory concentration ratio as a predictor of vancomycin treatment outcome in methicillin-resistant Staphylococcus aureus bacteraemia. Int J Antimicrob Agents. 2014 Feb;43(2):179-83. doi: 10.1016/j.ijantimicag.2013.10.017. Epub 2013 Nov 18.
3. Know when to use and be able to efficiently calculate:
  - Ideal body weight
  - Adjusted body weight
  - Creatinine clearance using the Cockcroft-Gault Equation
  - An estimated volume of distribution for vancomycin
  - An estimated Ke for vancomycin
  - An estimated half-life
  - The estimated maximum and minimum vancomycin concentration for a given patient and vancomycin dosing regimen

Your participation grade will be based upon the ***completion*** of the following:

Pre-preparation quiz (20%), individual in class quiz (10%), team in class quiz (20%), participation in the case discussion (30%), end of activity survey (20%)

To be best prepared to dose vancomycin you will need to:

1. Bring paper to write on and with

2. Bring a scientific calculator

3. Bring equation sheets

4. Review notes from kinetics course work

5. Review vancomycin dosing protocol

(Note: this last item will be published following your kinetics course content related to vancomycin. It will be aligned with your kinetics course content, and it will provide some specifics for what equations to use (for the day of class) to make sure we all get the same numbers when we’re working together in class)

## Appendix C. IRAT/TRAT

**Kinetics Team Based Learning Pre-Quiz**

EB is a 50 year old male.

Ht: 6′ 0”; Wt: 135 kg; SCr 1.1

1. Based on the reading by Rybak et al., vancomycin dose is generally calculated using
  - Total body weight
  - Adjusted body weight
  - Ideal body weight
  - Standard dose without regard to weight
2. Based on your preparation in the pharmacokinetics course work, what is EB’s CrCl?
  - 88.18 mL/min
  - 114.27 mL/min
  - 120 mL/min
  - 153.41 mL/min
3. Based on the reading by Rybak et al., what is the typical maximum infusion rate for vancomycin?
  - 250 mg per 30 min
  - 500 mg per 30 min
  - 750 mg per 30 min
  - 1000 mg per 30 min
4. Based on the reading by Rybak et al., what is the goal vancomycin trough in hospital acquired pneumonia?
  - 5-10 mcg/mL
  - 10-15 mcg/mL
  - 10-20 mcg/mL
  - 15-20 mcg/mL
5. Based on the reading by Rybak et al., vancomycin as a cause of nephrotoxicity is most related to
  - isolated supratherapeutic trough concentrations
  - longer treatment courses and significantly supratherapeutic trough concentrations
  - trough concentrations 15-20 mcg/mL and supratherapeutic trough concentrations
  - use at any level or concentration
6. Based on your preparation in the pharmacokinetics course work, what is EB’s estimated vancomycin half-life?
  - 8.88 h
  - 7 h
  - 6.66 h
  - 5.25 h
7. Based on your preparation in the pharmacokinetics course work, if EB was given vancomycin 1000 mg IV Q12H, what would be his predicted steady state peak be if the dose was given over 2 h and assuming VD = 0.7 L/Kg?
  - 15.12 mcg/mL
  - 16.12 mcg/mL
  - 17.12 mcg/mL
  - 18.12 mcg/mL
8. Based on your preparation in the pharmacokinetics course work, what is EB’s predicted trough?
  - 4.14 mcg/mL
  - 5.15 mcg/mL
  - 6.39 mcg/mL
  - 7.39 mcg/mL
9. Based on your preparation in the pharmacokinetics course work, how would this concentration change if EBs total body weight decreased?
  - Predicted trough concentration would increase
  - Predicted trough concentration would decrease
  - Predicted trough concentration would not change
  - Predicted trough concentration not related to total body weight
10. Based on the reading by Jung et al., if EB was your patient and he was being treated for a very serious infection would you wait to obtain a trough before the 4th dose before you adjusted his vancomycin dosing regimen?
  - Yes
  - No

## Appendix E. Post-TBL Perception Survey

**Post-Exercise Perception Survey**

**Instructions:** This evaluation instrument is to assess the frequency the team applied teamwork competencies to make a positive impact on the team process. Answer the questions for your team by filling in the bubble to indicate how frequently you think your team demonstrated the competency.

**Teamwork Competencies**

**Contributes to team meetings to achieve group tasks:** initiates, seeks and gives information clarifies, summarizes, takes consensus, and is accountable

**Maintains positive group communication:** serves as a gatekeeper, encourages, resolves conflict, acknowledges feelings, set standards, and is open

**Displays a positive attitude:** values team decisions, has positive regards and respect for all members, fosters mutual trust, open to feedback, shares team vision

**My team…**

	**Consistently**	**Regularly**	**Occasionally**	**Rarely**	**Never**
1. **Contributes**	**○**	**○**	**○**	**○**	**○**
2. **Maintains**	**○**	**○**	**○**	**○**	**○**
3. **Displays**	**○**	**○**	**○**	**○**	**○**

**Team Interdependence (these are all 5 point likert scale from strongly agree to strongly disagree).**

**The team worked best when we coordinated our work closely**	Strongly agree	Agree	Neutral	Disagree	Strongly disagree
**Team members had to work together to complete group tasks**	Strongly agree	Agree	Neutral	Disagree	Strongly disagree
**The way individual members performed their jobs had a significant impact on others in the team**	Strongly agree	Agree	Neutral	Disagree	Strongly disagree

**My ability to apply pharmacokinetic concepts in establishing a therapeutic regimen for vancomycin has improved**	Strongly agree	Agree	Neutral	Disagree	Strongly disagree
**My understanding of medications that are renally cleared has improved**	Strongly agree	Agree	Neutral	Disagree	Strongly disagree
**My understanding of how renal function and volume of distribution affects vancomycin dose has improved**	Strongly agree	Agree	Neutral	Disagree	Strongly disagree
**My understanding of linear pharmacokinetics has improved**	Strongly agree	Agree	Neutral	Disagree	Strongly disagree

## Appendix F. Vancomycin Dosing Protocol

**Pharmacokinetic Consult Service Guidelines: Vancomycin Protocol**

Standard vancomycin infusion time is 2 h

- For a different infusion time a specific order must be written
  ○ This is discourage when possible to limit errors in dosing and administration

Vancomycin doses may be ordered in multiples of 250 mg

- Maximum single dose is 2500 mg

Doses of 250 mg to 1250 mg are diluted in 250 mL unless otherwise ordered

Doses of 1500 mg to 2500 mg are diluted in 500 mL unless otherwise ordered

Vancomycin is diluted in Normal Saline unless specifically ordered otherwise

Vancomycin doses are not held pending lab results unless specifically ordered

Approved dosing intervals for vancomycin are 6, 8, 12, 24, 48 h

- Previously 18 or 36 h intervals were disallowed at Bull’s All Saints Hospital due to repeated administration errors

Pharmacy is permitted to order chemistry laboratories, blood counts, and vancomycin concentrations to monitor vancomycin therapy and renal function.

- Additional laboratories or medications require a physician order
- AM Labs are drawn at 4:00 AM
- Blood drawn with AM labs is typically enough volume to add on a vancomycin concentration if needed later the same day

Volume of distribution for all patients should be initially estimated as 0.7 L/Kg

**When calculating CrCl use IBW. Unless TBW is < IBW, then use TBW.**

**Note to students:**

In this scenario series, as in real life, you will have to respond to patient needs and dosing history that you may or may not have implemented. Therefore, as the scenario progresses you are stuck with what happened regardless of what you would have done.
